# Identification of genetic loci that control mammary tumor susceptibility through the host microenvironment

**DOI:** 10.1038/srep08919

**Published:** 2015-03-09

**Authors:** Pengju Zhang, Alvin Lo, Yurong Huang, Ge Huang, Guozhou Liang, Joni Mott, Gary H. Karpen, Eleanor A. Blakely, Mina J. Bissell, Mary Helen Barcellos-Hoff, Antoine M. Snijders, Jian-Hua Mao

**Affiliations:** 1 Life Sciences Division, Lawrence Berkeley National Laboratory, Berkeley, CA 94720; 2 NYU School of Medicine, New York, NY 10016

## Abstract

The interplay between host genetics, tumor microenvironment and environmental exposure in cancer susceptibility remains poorly understood. Here we assessed the genetic control of stromal mediation of mammary tumor susceptibility to low dose ionizing radiation (LDIR) using backcrossed F1 into BALB/c (F1Bx) between cancer susceptible (BALB/c) and resistant (SPRET/EiJ) mouse strains. Tumor formation was evaluated after transplantation of non-irradiated *Trp53*-/- BALB/c mammary gland fragments into cleared fat pads of F1Bx hosts. Genome-wide linkage analysis revealed 2 genetic loci that constitute the baseline susceptibility via host microenvironment. However, once challenged with LDIR, we discovered 13 additional loci that were enriched for genes involved in cytokines, including TGFβ1 signaling. Surprisingly, LDIR-treated F1Bx cohort significantly reduced incidence of mammary tumors from *Trp53*-/- fragments as well as prolonged tumor latency, compared to sham-treated controls. We demonstrated further that plasma levels of specific cytokines were significantly correlated with tumor latency. Using an *ex vivo* 3-D assay, we confirmed TGFβ1 as a strong candidate for reduced mammary invasion in SPRET/EiJ, which could explain resistance of this strain to mammary cancer risk following LDIR. Our results open possible new avenues to understand mechanisms of genes operating via the stroma that affect cancer risk from external environmental exposures.

An unavoidable consequence of living in the natural world and modern industrial societies is the pervasive exposure to low dose ionizing radiation (LDIR) and chemical carcinogens. Radiation exposures from diagnostic medical devices and air travel have increased substantially in the last decades; indeed the annual exposure for the American public has doubled over the last 20 years^1^. Major problems in assessing human cancer risk to LDIR, defined as less than or equal to 10 cGy, are that humans are genetically heterogeneous, exposure is pervasive and there are no meaningful assays for assessing differences in susceptibility. Thus, efforts to detect genetic contributions to LDIR-induced cancer development and progression in human populations are challenging, and whether LDIR is a risk factor for cancer remains controversial^2^,^3^,^4^,^5^,^6^. Studies in mouse strains that exhibit different susceptibilities to environmentally-induced cancers offer unprecedented opportunities to identify the primary genetic loci and investigate their mechanistic contributions. Moreover, these experimental studies afford precise exposures, standardized husbandry to control for other environmental components of risk, and comprehensive analysis of phenotypes.

Cancer is genome and organ specificity (epigenome) gone awry since many properties that are regulated in the context of the physiology of a complex organism are altered, all of which play important roles in tumor induction and progression. The pathophysiology of cancer, as that of any other organ, depends not only on the intrinsic properties of the parenchymal component (tumor cells), but also on other organismic compartments including stroma^7^, extracellular matrix integrity^8^, and the immune, endocrine and vascular systems^9^. Consequently, cancer susceptibility to radiation, specially the role of low dose, is determined by canonical factors traditionally measured only in tumor cells, such as mutation, proliferation, apoptosis, and DNA repair, but is also influenced by the tumor cell's microenvironment, including immune responses^10^,^11^. Radiation-induced changes in the stromal microenvironment can contribute to malignant progression *in vivo*^12^,^13^. Furthermore, the two main compartments of a tissue (epithelia and stroma) are not independent, but rather there is continuous dynamic reciprocity between cells and their surroundings, such that signaling of the extrinsic factors determines intrinsic cellular activity, and vice versa. Importantly, radiation can affect both compartments^14^,^15^,^16^. In this study, we employed a mouse mammary chimera model to identify the genetic loci that act through the stromal microenvironment to regulate mammary tumor susceptibility to LDIR. In the radiation chimera model, inguinal mammary glands are cleared of endogenous epithelia, the hosts are irradiated or sham-treated, and subsequently transplanted with a *Trp53*-/- epithelium, which makes them more susceptible to oncogenic transformation. Mammary cancer develops over the course of a year at a high rate due to the absence of *Trp53* function^13^. In a previous study, LDIR (10 cGy of X-ray) increased the incidence and decreased the latency of *Trp53* null mammary tumors in inbred BALB/c mice^13^.

Radiation exposure enhances the susceptibility of the BALB/c inbred mouse strain to develop mammary cancer^17^,^18^,^19^. In contrast, SPRET/EiJ mice, which only recently have been inbred, are resistant to cancer induced by chemical carcinogens, radiation or other environmental factors^20^,^21^,^22^,^23^. Backcrosses between *Mus spretus* and *Mus musculus* strains have proven useful for identifying single nucleotide polymorphisms (SNPs) associated with diseases^20^,^23^. Here we combined the radiation chimera model^12^,^13^ with systems genetics to study the contribution of host genetic variations that affect stromal microenvironments and systemic responses in cancer risk after exposure to LDIR.

## Results

### Study design for a systems genetics analysis of stromal microenvironment in mammary tumor susceptibility to LDIR

**Figure 1:**
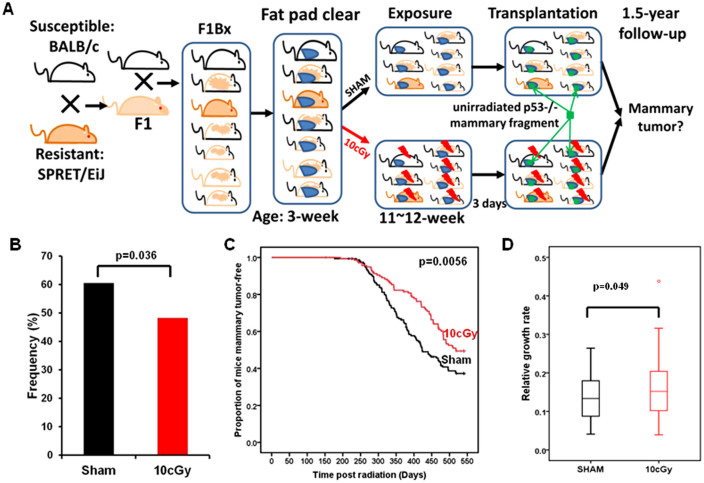
Effect of LDIR on tumor phenotypes in genetically diverse F1Bx hosts. (A) Study design for a systems genetics analysis of mammary tumor susceptibility after low dose radiation exposure. (B–D) 10 cGy radiation of mice (B) reduced the incidence of mammary tumors significantly (death without tumors), (C) delayed their time of appearance, and (D) increased tumor growth. The p-values were obtained from Fisher exact test in (B), log rank test in (C), and non-parametric Mann-Whitney U test in (D).

To examine the effects of LDIR and the stromal microenvironment on mammary tumor development, the endogenous epithelium was surgically removed from F1Bx female mammary glands at 3-weeks of age, and at 11 ~ 12 weeks, mice were either irradiated whole-body with 10 cGy X-rays (LDIR) or sham treated (Figure 1A). Three days post irradiation, un-irradiated inguinal mammary gland fragments from BALB/c *Trp53* null (p53-/-) mice were transplanted into the LDIR- and sham-treated F1Bx hosts (Figure 1A). Mice were monitored for tumor development by palpation for 18 months. Upon detection, tumor growth rate was determined by measuring tumor volume at each week (see Methods). To measure cytokine levels in plasma, blood was collected from all mice by orbital bleeds at 6 hours and 15 weeks after radiation exposure.

### Mammary tumor development in genetically diverse hosts is reduced by LDIR

In a previous study, LDIR exposure of inbred susceptible BALB/c hosts decreased tumor latency and increased the frequency of tumor incidence for implanted *Trp53* null epithelia^13^. Surprisingly, we found that the frequency of *Trp*53 null tumors in LDIR treated F1Bx hosts was reduced by 12.3% (p = 0.036) compared to sham-treated hosts by the time the experiment was terminated (Figure 1B). Moreover, whole body irradiation of F1Bx host significantly delayed tumorigenesis (Figure 1C). However, once the tumors were formed, the rate of tumor growth (see Methods) was increased in irradiated F1Bx host mice relative to sham (Figure 1D), as reported previously^13^. Over 80% of tumors were estrogen receptor (ER) positive and this frequency was not altered by host irradiation (p = 0.37; Figure S1A). The majority of tumors were adenocarcinoma (~50%) or squamous cell carcinoma (~40%), the remaining tumors were spindle cell carcinoma, and the distribution of histological types was unaffected by host irradiation (p = 0.13; Figure S1B). We conclude that introgression of the SPRET/EiJ genome with BALB/c resulted in reduced frequencies and increased latency of mammary tumors in low dose irradiated hosts.

### Identification of genetic loci that control mammary tumor latency

**Figure 2:**
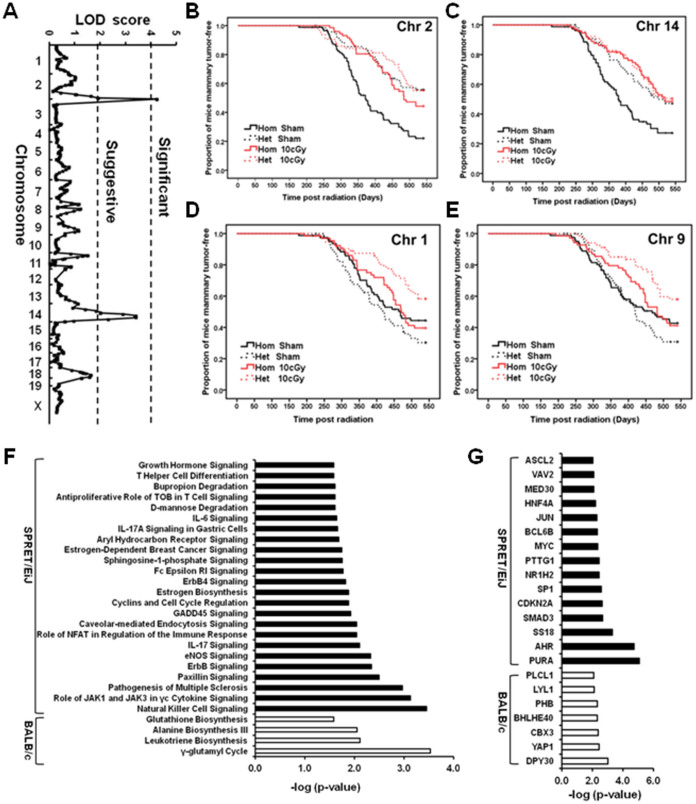
Identification of genetic loci that control tumor latency. (A) Genome-wide LOD scores for tumor latency of Sham-treated mice were obtained by R/QTL. (B–E) Kaplan-Meier curves for tumor latency at the locus of Chromosome 2 (B), 14 (C), 1 (D), and 9 (E). The p-values were obtained by log rank test. (F) Ingenuity Pathway Analysis (IPA) revealed the potential signaling pathways that were enriched among the candidate genes located within the identified loci. (G) Ingenuity Pathway Analysis (IPA) revealed upstream transcriptional factors that regulate the expression of the candidate genes located within the identified loci.

We devised our system analysis of susceptibility to determine how the genetics of the host affects mammary tumor development and progression of *Trp53* null epithelial fragments in F1Bx mice. We interrogated the genetic loci associated with tumor latency and frequency by genome-wide genotyping using Illuminal SNP microarrays. Only two loci, one on chromosome 2 (LOD score = 4.26) and the other on chromosome 14 (LOD score = 3.43) were associated with tumor latency in sham-treated mice (Figure 2A to 2C). Mice with homozygous BALB/c alleles at these two loci had significantly shorter latency than those that were heterozygous (one allele from BALB/c and one from SPRET/EiJ) (Figure 2B and 2C), suggesting that some SPRET/EiJ genes delay tumor development in exogenously-grafted BALB/c *Trp53* null epithelial fragments. In contrast, we identified 15 genetic loci that interact with LDIR exposure to control tumor latency (Table 1). The SPRET/EiJ allele in 11 of these 15 loci confers reduced risk to tumor development after host exposure to LDIR (Figure 2D and 2E, Table 1), whereas 4 remaining SPRET/EiJ alleles confer increased susceptibility. These results suggest that host genetic variants strongly influence mammary cancer latency after exposure to LDIR. The observation that many more genetic loci were found in the LDIR cohort compared to the sham cohort suggests a strong host genetic contribution specific to radiation. Given that the host, but not cells producing the tumor, were irradiated, the genetic contribution to mammary tumor susceptibility involves non-cell autonomous mechanisms, which are clearly through host microenvironment.

To further explore genetic associations with cancer risk after exposure to LDIR, we discovered 696 candidate genes located within 5 Mb of the peak of the identified loci (Table S1) and used Ingenuity Pathway Analysis (IPA) to examine pathway enrichments. Of these, 185 genes were within 4 loci on chromosomes 2, 11, 14, and 16, where homozygous BALB/c alleles are associated with increased cancer latency after exposure to LDIR, and were enriched in four pathways, γ-glutamyl cycle, leukotriene biosynthesis, alanine biosynthesis III and glutathione biosynthesis (Figure 2F). In contrast, 511 genes were within 11 regions where the heterozygous SPRET/EiJ allele is associated with increased latency after LDIR treatment, and were enriched for 24 pathways (Figure 2F). Importantly these 11 loci were enriched for genes involved in regulating the immune response including signaling pathways of natural killer cells, cytokines, etc. (Figure 2F). Analysis of the upstream regulators of these candidate genes indicated that the TGFβ (SMAD3) and p53 (CDKN2A) pathways are likely to be involved in mammary tumor susceptibility in response to LDIR (Figure 2G).

### Association of plasmas cytokine levels with tumor latency

**Figure 3:**
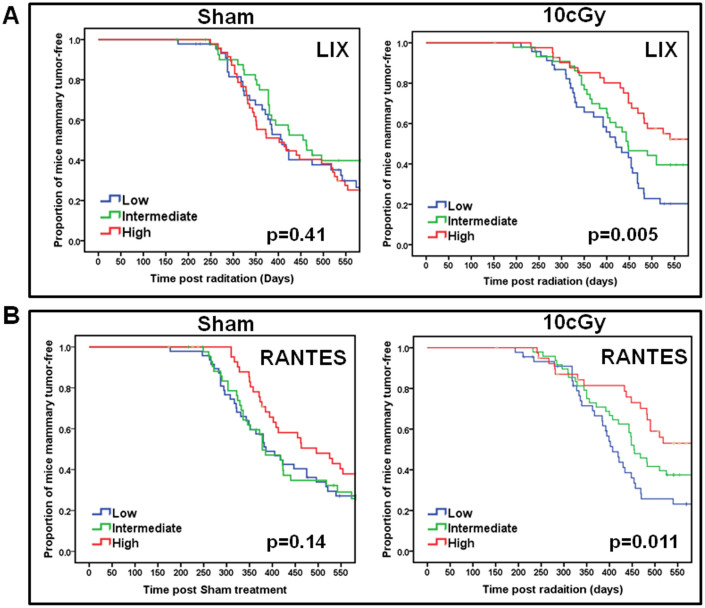
Association of plasma cytokine levels with tumor latency. The impact of plasma levels of LIX (A) and RANTES (B) on tumor latency is observed in 10 cGy treated mice, but not Sham treated mice. The p-values were obtained by log rank test.

Given the strong representation of cytokine signaling pathways within the identified stromal genetic loci, we assessed the association of plasma cytokine levels with tumor latency by dividing mice into three groups based on plasma cytokine levels (low = bottom third, moderate = middle third, and high = top third). In sham-treated F1Bx mice, plasma levels of eotaxin at 6 hrs after treatment and IL-1A at 15 weeks after treatment were significantly associated with tumor latency (Figure S2). In 10 cGy-treated F1Bx mice, plasma levels of two cytokines (G-CSF and IL-13) at the early time point and three cytokines (IP10, LIX, and RANTES) at the later time point were significantly associated with tumor latency (Figure 3, Figure S3). For example, mice with high levels of LIX (Figure 3A) or RANTES (Figure 3B) developed tumors significantly later than those with low levels. These data are consistent with the demonstration that 10 cGy treatment significantly increases RANTES levels (Chang et al., submitted).

### Mammary gland development differs in BALB/c and SPRET/EiJ mice

**Figure 4:**
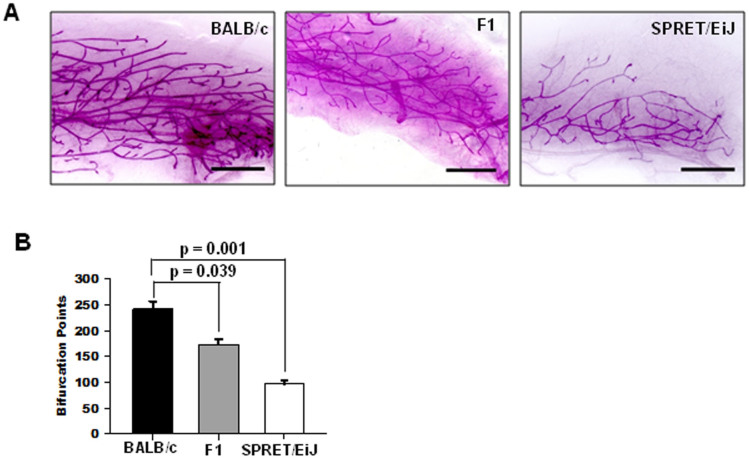
Effect of host genetic background on mammary gland architecture. (A) Whole-mount imaging mammary gland by optical microscopy. Mammary glands were collected at 10 weeks after birth from BALB/c, SPRET/EiJ, and F1 hybrids between BALB/c and SPRET/EiJ. (B) Quantification of mammary gland branching. The p-values were obtained by t-test.

To elucidate functional mechanisms of these mammary cancer susceptible loci, we further examined whether BALB/c and SPRET/EiJ parental strains display any differences in normal mammary development. Mammary glands were collected from BALB/c and SPRET/EiJ mice at different ages, then stained with carmine alum to visualize the spatial arrangement of their ductal tree in whole mounts (Figure S4). We found that SPRET/EiJ mammary glands have fewer branches in comparison to BALB/c mammary glands (Figure 4A, Figure S4), further confirmed by quantitative analysis of the area occupied by epithelial cells (Figure S5) and branching (Figure 4B) at 10 weeks after birth. The morphology of mammary glands from F1 hybrids between BALB/c and SPRET/EiJ mimicked SPRET/EiJ (Figure 4), suggesting that the SPRET/EiJ genome is dominant for this phenotype.

### TGFβ1 signaling regulates stromal invasion in mammary organoids

**Figure 5:**
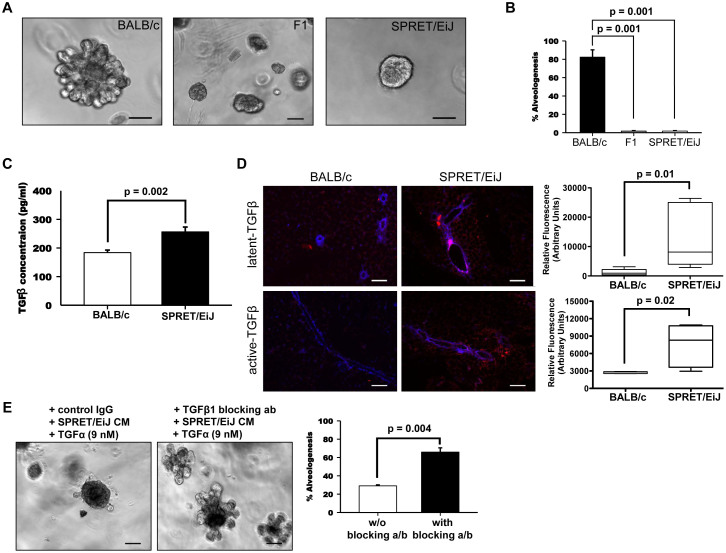
TGFβ1 regulates growth factor-induced branching. (A) Representative phase-contrast images of BALB/c or SPRET/EiJ organoids induced to branch with TGFα in growth factor-reduced Matrigel. Organoids were grown for 5 days after treatment. (B) Quantification of the number of BALB/c and SPRET/EiJ organoids in each condition that had three or more branches (n = 6 experiments, >200 organoids/condition). (C) TGFβ1 levels after treatment with TGFα. ELISA analysis of culture media harvested from organoid cultures (n = 6 independent experimental sets). (D) Merged channel images BALB/c and SPRET/EiJ mammary gland sections stained with DAPI, latent-TGFβ1 and active-TGFβ1 (n = 6 experiments). (E) Representative phase-contrast images of BALB/c organoids induced to branch with TGFα. After 24 hours, organoids were treated with SPRET/EiJ culture media either alone or with TGFβ1 blocking antibody and grown for 5 days. Quantification of the number of BALB/c organoids in each condition that had three or more branches (n = 6 experiments, >100 organoids/condition). The p-values were obtained by t-test.

Parental strain differences in branching and invasion into the fat pad during mammary development led us to investigate which regulators of these physiological processes differ between BALB/c and SPRET/EiJ. Our SNP mapping and bioinformatic analysis identified the TGFβ pathway, a known regulator of mammary branching and development^24^, as a potential mediator of mammary tumor susceptibility. We further examined the involvement of TGFβ1 signaling on breast cancer risk using an *ex vivo* organoid culture model to assess stromal invasion^25^. Ductal fragments from BALB/c, SPRET/EiJ and their F1 hybrid mammary glands were cultured in growth factor-reduced Matrigel (Figure 5A), and the number of branching organoids were quantified (Figure 5B). We observed that SPRET/EiJ and F1 hybrid organoids were unable to form any branched structure, whereas BALB/c organoids branched as reported previously; similar results were obtained using standard 3D culture conditions in 3 mg/ml collagen I (Figure S6). These results suggest that SPRET/EiJ and BALB/c epithelial cells sense and respond to their microenvironments differently.

We also observed that TGFβ1 concentrations measured in culture media were significantly higher when isolated from the SPRET/EiJ organoids compared to BALB/c, suggesting a more direct link between TGFβ1 levels and branching inhibition (Figure 5C, Figure S7). Furthermore, assessment of the localization of active and inactive TGFβ1 in developing mammary glands showed a significant increase in active TGFβ1 in the SPRET/EiJ mammary glands compared to BALB/c (Figure 5D). Finally, addition of SPRET/EiJ culture media inhibited branching of BALB/c organoids, and the effect was significantly reduced after treatment with a TGFβ1 blocking antibody (Figure 5E). These results support the idea that higher TGFβ1 levels in SPRET/EiJ restrict branching, possibly leading to protection against radiation-induced cancers. Further studies are required to determine the precise relationship between *ex vivo* ductal branching and tumor susceptibility.

## Discussion

Controversy exists in the literature regarding the effects of exposure of humans and experimental animal models to LDIR. Part of the controversy is likely due to our incomplete understanding of the genetic control of a number of important physiological systems that are key to radiation responses. In this study, we developed a model system to investigate how genetic variation affects the tumor susceptibility of transplanted *Trp53* null mammary epithelia in response to irradiation through the host microenvironment. In this model system, genetically identical and oncogenically primed *Trp53* null mammary cells from inbred BABL/c mice were transplanted into genetically heterogeneous hosts (F1Bx mice) to separate radiation effects on promotion via stroma from direct initiation via DNA damage. We showed that LDIR *reduced* the incidence and *increased* the latency of *Trp53* null mammary tumors in F1Bx hosts. These results differ from previous observations in inbred BALB/c mice, where 10 cGy increased the incidence and decreased the latency of *Trp53* null mammary tumors^13^. We conclude that the contribution of host microenvironment to cancer risk in mice following exposure to LDIR strongly depends on host genetic backgrounds.

Our studies demonstrate that genetic variations, likely acting both systemically and stromally, regulate the mammary microenvironment, which subsequently influences mammary cancer development. We identified 15 host genetic loci that control tumor latency, the majority of which (13 loci) were associated only with tumor latency after exposure to LDIR. This indicates a strong interaction between host genetics and LDIR in mammary tumor susceptibility. Surprisingly, 13 of 15 loci are also close to tumor susceptibility loci previously reported in other studies (Table S1). The majority of these studies involved chemically-induced tumorigenesis^21^,^26^, suggesting common regulators of susceptibility between different types of environmental exposures. More interesting is the fact that the genetic loci identified in our study control host microenvironment, indicating that stromal microenvironment could also play an important role in chemical carcinogens susceptibilities.

Radiation-induced activation of pathways that control release of inflammatory cytokines varies among mouse strains^27^,^28^,^29^ and may contribute to genetic susceptibility to radiation induced leukemia in mice^27^. Bioinformatics analysis shows that the genetic loci identified in this study are enriched for genes involved in regulating the immune response, including signaling pathways of natural killer cells and cytokines. Consistent with this, we observed significant differences in plasma cytokine levels between SPRET/EiJ and BALB/c mice in response to LDIR, and found that differences in plasma cytokine levels in F1Bx mice are controlled by genetic variation (Chang et al., submitted), some of which correspond to loci identified in this study as regulating tumor frequency and latency. Moreover, certain plasma cytokine levels correlate with mammary tumor development after exposure to LDIR. These results indicate that the mammary tumor susceptibility to LDIR, at least in part, is controlled through the regulation of cytokines, although additional studies are required to identify the direct causes and consequences of cytokine levels and their genetic regulation.

Analysis of the upstream regulators of candidate genes within these loci revealed the TGFβ pathway as a potential mediator of mammary tumor susceptibility to LDIR. A number of studies have demonstrated that TGFβ1 plays a critical role in radiation-induced breast cancer risk^15^,^30^,^31^. It has been proposed that TGFβ1 acts as a tumor suppressor at early stages of tumor outgrowth, either by suppressing growth and/or inducing differentiation^32^,^33^,^34^,^35^,^36^. In this study, we found that compared to BALB/c, SPRET/EiJ mice have significantly higher TGFβ1 levels. Importantly, we demonstrate that the reduced branching and invasion observed in parental SPRET/EiJ mammary glands and *ex vivo* hybrid organoids requires high TGFβ1 levels, consistent with previous reports that the use of TGFβ1 pathway inhibits invasion and branching in 3D stromal collagen gels^24^. This could also explain resistance of SPRET/EiJ strain to tumor development in general, and here to mammary cancer risk following LDIR.

In summary, we used a comprehensive systems biology approach to identify genetic variations that control mammary cancer susceptibility by altering host responses to LDIR. We show that these genetic variations actually prevent or retard the effect of radiation to promote malignancy through host microenvironmental changes and/or systemic effects, including control of cytokines such as TGFβ1 levels. We propose that human homologues of these genes could be used to establish susceptibility in individuals, and may identify preventive strategies to identify the populations that are at higher risk for cancer.

## Methods

### Mice

Animal treatment and care was carried out in accordance with the animal protocols and approved by the Animal Welfare and Research Committee at Lawrence Berkeley National Laboratory. SPRET/EiJ mice were obtained from Jackson Laboratories (Bar Harbor, Maine). The female interspecific F1 hybrid mice between BALB/c, a strain susceptible to radiation-induced mammary tumor development and SPRET/EiJ, a resistant strain, were crossed with male BALB/c to generate F1 backcross (F1Bx) mice (BALB/c × SPRET/EiJ) × BALB/c. The inguinal fat pad of F1Bx host mice (n = 350) were divested of endogenous mammary epithelium at 3 weeks of age At 10–11 weeks of age, half of the mice received 10 cGy of whole-body radiation 3 days prior to transplantation of the inguinal mammary glands with *Trp53* null fragments. Mice were monitored and palpated for mammary tumor development for 18 months. Upon detection, tumor growth was determined with digital calipers, and volume was estimated every week by the known formula: *Tumor volume = length × width*^*2*^* × 0.5*. We transformed the tumor volume logarithmically, and estimated a linear regression curve with time for each tumor. The slope of the line was defined as the rate of tumor growth.

### Mammary gland tissue collection and wholemounts

At the time of dissection, the stage of estrous was determined by vaginal lavage followed by cytological analysis. For each study, the thoracic and inguinal mammary glands were excised and frozen immediately in freezing medium (90% fetal bovine serum and 10% dimethyl sulfoxide) for organoid cultures, banked in optimum cutting temperature (OCT) compound (Tissue-Tek) for immunohistochemistry, or they were formalin fixed for histological analysis. One inguinal gland was fixed in Carnoy's solution for 30 minutes then stained with carmine alum overnight to analyze ductal/alveolar morphology. Histomorphometry to compare differences in epithelial density was performed using a mammary wholemount. Quantification of epithelial density was done via an image processing and analysis program (ImageJ; National Institutes of Health). To quantify bifurcation points, each branch beginning from the nipple were counted on each mammary gland wholemount. H&E staining were generated by the UCSF Helen Diller Family Comprehensive Cancer Center Mouse Pathology Core.

### Plasma Cytokine Assay

Blood was collected by retro-orbital bleed from mice at 6 hr and 15 weeks after LDIR or sham- treatment in EDTA coated collection tubes and was processed by centrifugation at 1500 g at 4°C in a refrigerated microfuge for 10 min. Plasma was transferred and aliquoted to a fresh RNase/DNase free 1.5 microfuge tube. Plasma samples were stored at −80°C for cytokine profiling. Plasma diluted 1:1 was run in triplicate on 32 premixed Milliplex™ Cytokine Kit plates (Millipore) following the standard protocol. Samples were incubated overnight, processed and run on a Luminex 200™. 32 mouse cytokines and chemokines include: Eotaxin, G-CSF, GM-CSF, IFNγ, IL-1α, IL-1β, IL-2, IL-3, IL-4, IL-5, IL-6, IL-7, IL-9, IL-10, IL-12 (p40), IL-12 (p70), IL-13, IL-15, IL-17, IP-10, KC, LIF, LIX, MCP-1, M-CSF, MIG, MIP-1α, MIP-1β, MIP-2, RANTES, TNFα, VEGF.

### Genotyping

DNA was extracted from tail snips and DNA concentrations were measured with the Nanodrop ND-1000 Spectrophotometer and PicoGreen double-stranded quantification (Molecular Probes). The genome-wide scan was carried out at the Human Genome Core of UCSF. Illumina's Mouse Low Density Linkage panel Assays were used to genotype 350 mice at 377 SNPs.

### Determination of secreted TGFβ

Organoids were grown in lrECM three-dimensional culture and seeded at 200 per well in a 48-well plate in serum-free media. Cells were treated with TGFα for 48-hours and the amounts of total and active TGFβ were determined using a commercial TGFβ1 enzyme-linked immunosorbent assay (ELISA) kit (Invitrogen).

### In vitro branching morphogenesis assays

Three-dimensional primary cultures were generated as previously described^25^. Briefly, to generate organoids, we embedded 200 purified epithelial mammary fragments in 200 μl of growth factor-reduced Matrigel (BD Biosciences) or 3 mg ml^−1^ collagen-1 gels (BD Biosciences) and stimulated with 9 nM TGFα (Sigma). For TGFβ1 blocking antibody experiments, three-dimensional cultures were treated everyday post seeding with collected culture media from SPRET/EiJ three-dimensional cultures with the addition of 9 nM TGFα (Sigma) and either 100 μg ml^−1^ of a rabbit polyclonal antibody that binds TGFβ (R&D Systems AB-100-NA), or with 100 μg ml^−1^ IgG control (Acris Antibodies AM03095AF-N).

### Immunofluorescent staining

Serial formalin fixed paraffin tissue sections (thickness: 5 μm) of mammary glands were generated by the UCSF Helen Diller Family Comprehensive Cancer Center Mouse Pathology Core. Mammary sections were deparaffinized and rehydrated in graded alcohol to 1X PBS, followed by heat-mediated antigen retrieval in Tris-EDTA Buffer (10 mM Tris Base, 1 mM EDTA, 0.05% Tween-20, pH 9.0) using a vegetable steamer. Slides were then permeabilized with 0.2% Triton-X and blocked for 1 hour in 10% serum. Slides were incubated overnight with either a polyclonal chicken antibody against active TGFβ1 antibody (R&D Systems AF-101-NA, 10) or a polyclonal goat antibody against LAP TGFβ1 (R&D Systems AB-246-NA, 50). Secondary antibodies used were rabbit anti-chicken 568 or 488 and rabbit anti-goat 488 or 568 (Invitrogen). Tissues were imaged on a Zeiss LSM 710 confocal microscope using a 0.45 NA × 10 air objective. Quantification was done as previously described^37^.

### Western blotting

Mouse organoids were lysed in 2% SDS/PBS. 30 μg of protein of each lysate was then separated on a Tris-glycine 4–20% gel. phospho-p44/42 was probed with a mouse monoclonal antibody (Cell Signaling 9106, 1:250), p44/42 was probed with a rabbit polyclonal antibody (Cell Signaling 9102, 1:1000), phospho-SMAD2 was probed with a rabbit polyclonal antibody (Cell Signaling 3104), and SMAD2 was probed with a rabbit monoclonal antibody (Cell Signaling 5339). The blot was stripped and re-probed with a rabbit polyclonal antibody against α-tubulin, used here as a loading control (Abcam ab18251, 1:1000).

### Statistical and linkage analysis

The Kaplan–Meier and Cox regression method was used to compare the tumor latency while the Chi-square test was used to compare mammary tumor frequency between 10 cGy or sham-treated mice. Statistical analysis was performed using SPSS version 12.0 (SPSS, Chicago, IL). Linkage analysis was carried out using R/QTL.

## Author Contributions

M.J.B., M.H.B.H. and J.H.M. provided conception and overall experimental design. P.Z., A.L., Y.H., G.H., G.L., J.M., E.A.B. and A.M.S. performed experiments. P.Z., A.L., A.M.S. and J.H.M. analyzed data. P.Z., A.L. and J.H.M. prepared all figures. G.H.K., M.J.B., M.H.B.H. and J.H.M. wrote the manuscript. All authors contributed to manuscript editing.

## Supplementary Material

Supplementary Information Supplementary Figures and Table

## Figures and Tables

**Table 1:** Genetic loci intact with LDIR controlling susceptibility to mammary tumor development

Chromosome	Location (Mb)	SNP ID	Homozygous				Heterozygous			
			p-value	HR*	95% CI for HR#		p-value	HR	95% CI for HR	
					Lower	Upper			Lower	Upper
1	174.28	rs13476248	0.97	0.99	0.64	1.54	1.86E-04	0.42	0.27	0.66
2	154.20	rs6376291	2.19E-04	0.47	0.32	0.70	0.88	0.96	0.57	1.61
3	33.93	rs6371982	0.76	0.89	0.44	1.83	2.70E-03	0.59	0.41	0.83
3	148.94	rs3657112	0.60	1.20	0.61	2.38	7.43E-04	0.54	0.38	0.77
4	8.73	rs13477549	0.41	0.83	0.53	1.29	3.09E-03	0.51	0.33	0.80
5	93.65	CEL-5_93652588	0.32	0.81	0.53	1.23	3.92E-03	0.50	0.31	0.80
7	114.58	rs13479513	0.80	0.91	0.44	1.88	2.52E-03	0.58	0.41	0.83
8	56.55	rs3685424	0.99	1.00	0.44	2.28	1.83E-03	0.58	0.41	0.82
9	55.40	rs13480208	0.69	0.92	0.60	1.40	4.07E-04	0.43	0.27	0.68
11	73.72	rs13481099	3.14E-03	0.52	0.33	0.80	0.36	0.81	0.52	1.27
12	96.44	rs3700012	0.36	0.81	0.52	1.27	0.48	0.85	0.55	1.33
14	78.69	rs3708535	6.81E-04	0.48	0.31	0.73	0.57	0.87	0.55	1.40
15	72.19	rs13482641	0.57	0.87	0.55	1.40	1.66E-03	0.56	0.39	0.80
16	50.66	rs4186129	4.34E-03	0.54	0.35	0.83	0.38	0.81	0.50	1.30
17	46.77	rs13483012	0.87	0.94	0.47	1.91	2.83E-03	0.59	0.41	0.83

*HR = Hazard Ratio for 10 cGy vs Sham.

^#^**CI = Confidence Interval.**
