# Supplementary material for: Identification of genetic loci that control mammary tumor susceptibility through the host microenvironment
Source: Sci Rep. 2015 Mar 9;5:8919. doi: 10.1038/srep08919 (PMC4352890; doi:10.1038/srep08919)
Supplement: Supplementary Information — Supplementary Figures and Table [file srep08919-s1.pdf]

Identification of genetic loci that control mammary tumor susceptibility through the host microenvironment

Pengju Zhang1#*, Alvin Lo1#, Yurong Huang1, Ge Huang1*, Guozhou Liang1, Joni Mott1, Gary H Karpen1, Eleanor A Blakely1, Mina J Bissell1, Mary Helen Barcellos-Hoff 2, Antoine M Snijders1, Jian-Hua Mao1‡

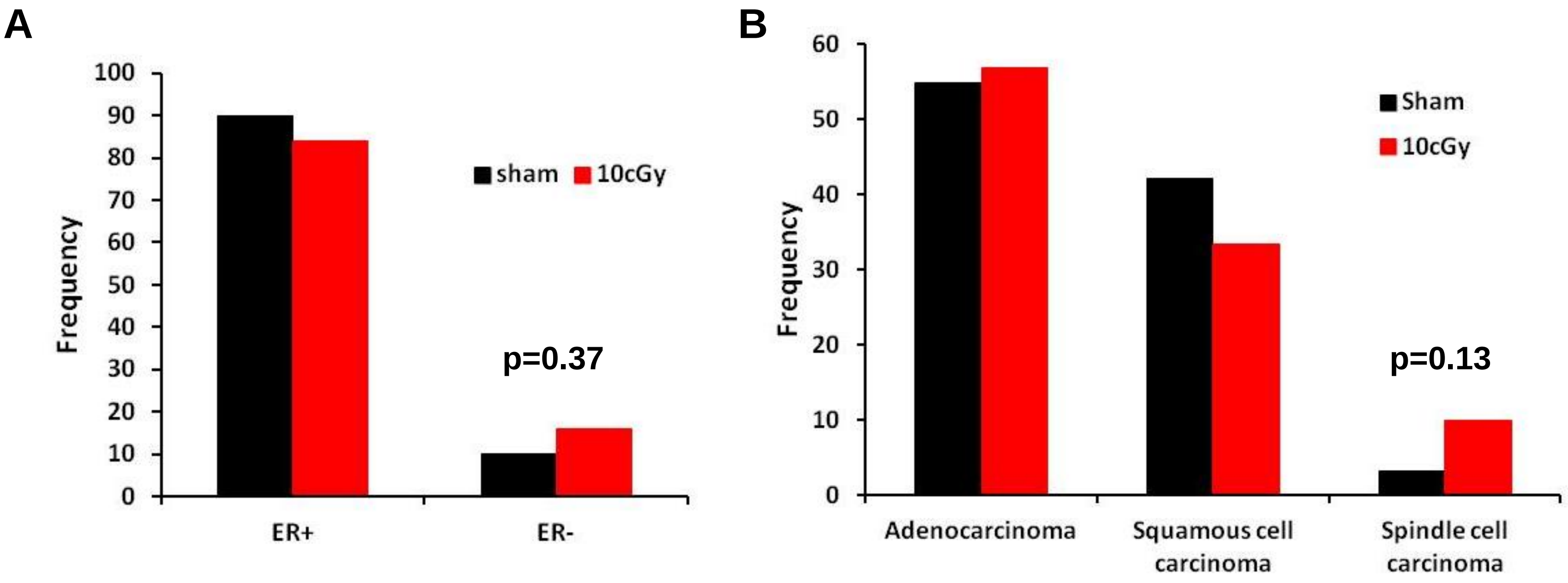

Figure S1. There is no effect of LDIR on tumor ER status (A) and pathological types (B) in genetically diverse F1Bx hosts. The p-value was obtained from Fisher exact test in (**A**) and Chi-square test in (**B**)

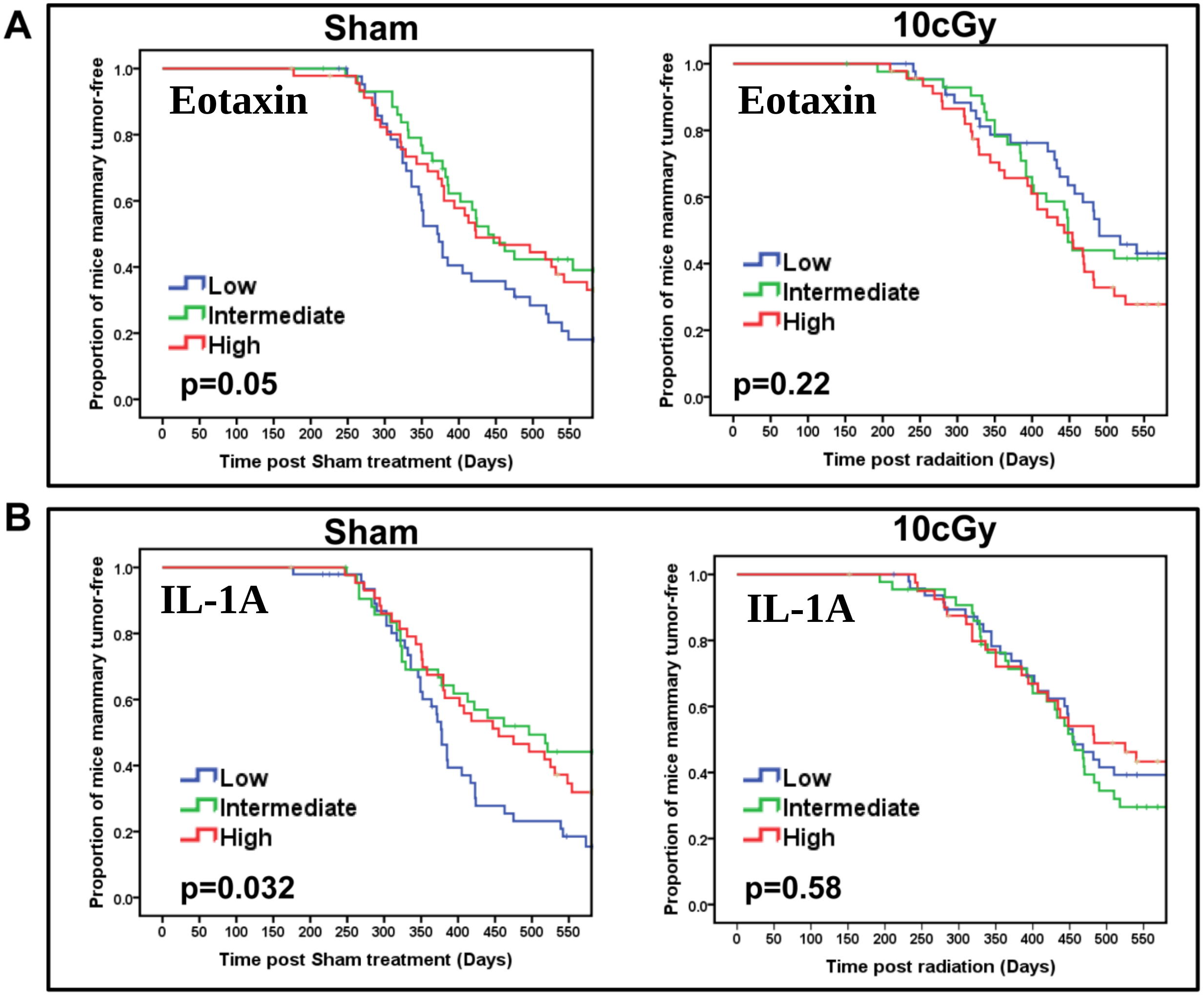

Figure S2. Association of plasma cytokine levels with tumor latency in Sham treated mice.

Impact of plasma levels of **(A)** Eotaxin at early time point (6 hrs post treatment) and **(B)** IL-1A at a later time point (15 weeks post treatment) on tumor latency in Sham treated mice, but no impact in 10cGy treated mice. The p-values were obtained by log rank test.

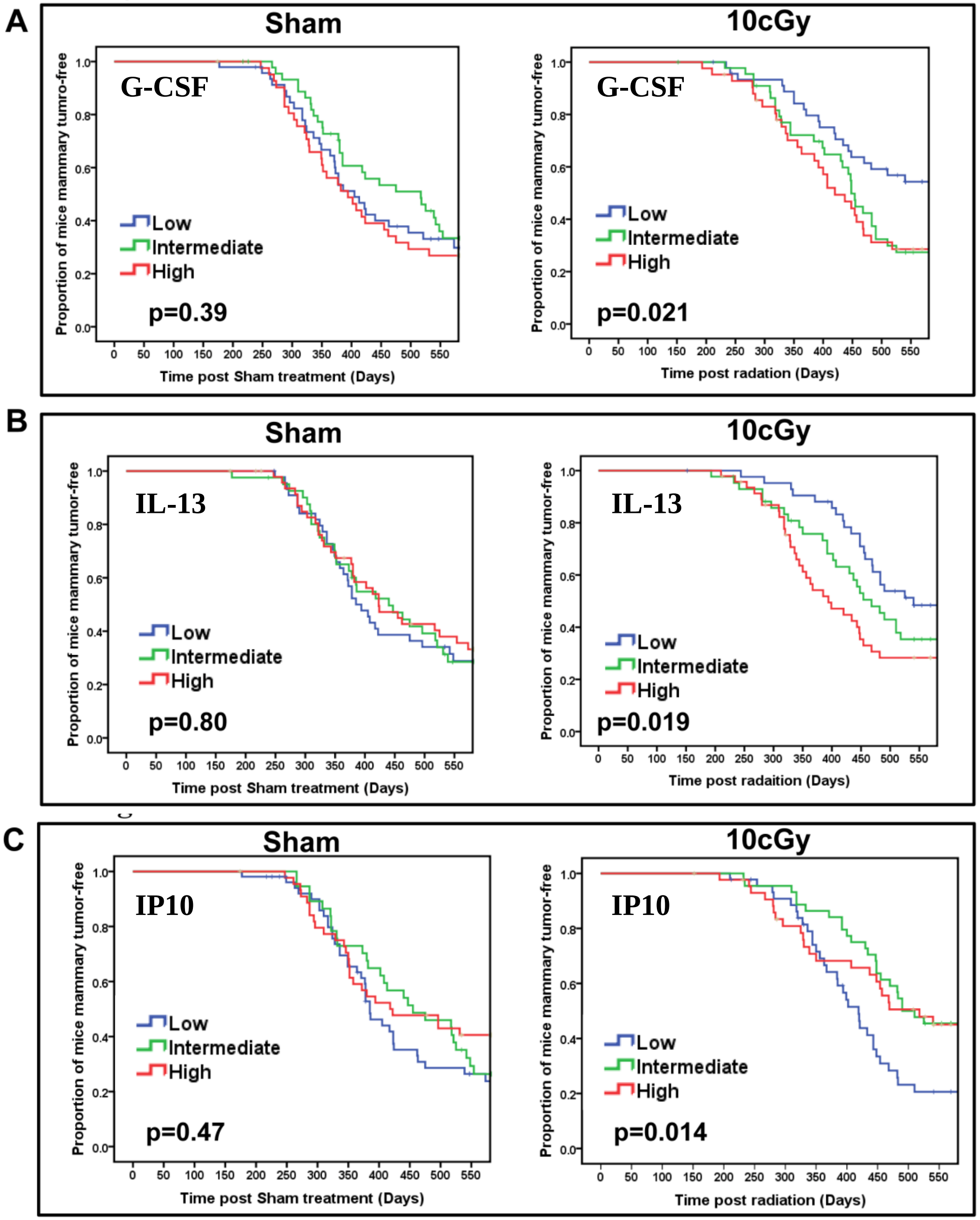

Figure S3. Association of plasma cytokine levels with tumor latency in 10 cGy treated mice.

There is the impact of plasma levels of **(A)** G-CSF and **(B)** IL-13 at the early time point (6 hrs post treatment) and **(C)** IP10at a later time point (15 weeks post treatment) on tumor latency in 10cGy treated mice, but no impact in Sham treated mice. The p-values were obtained by log rank test.

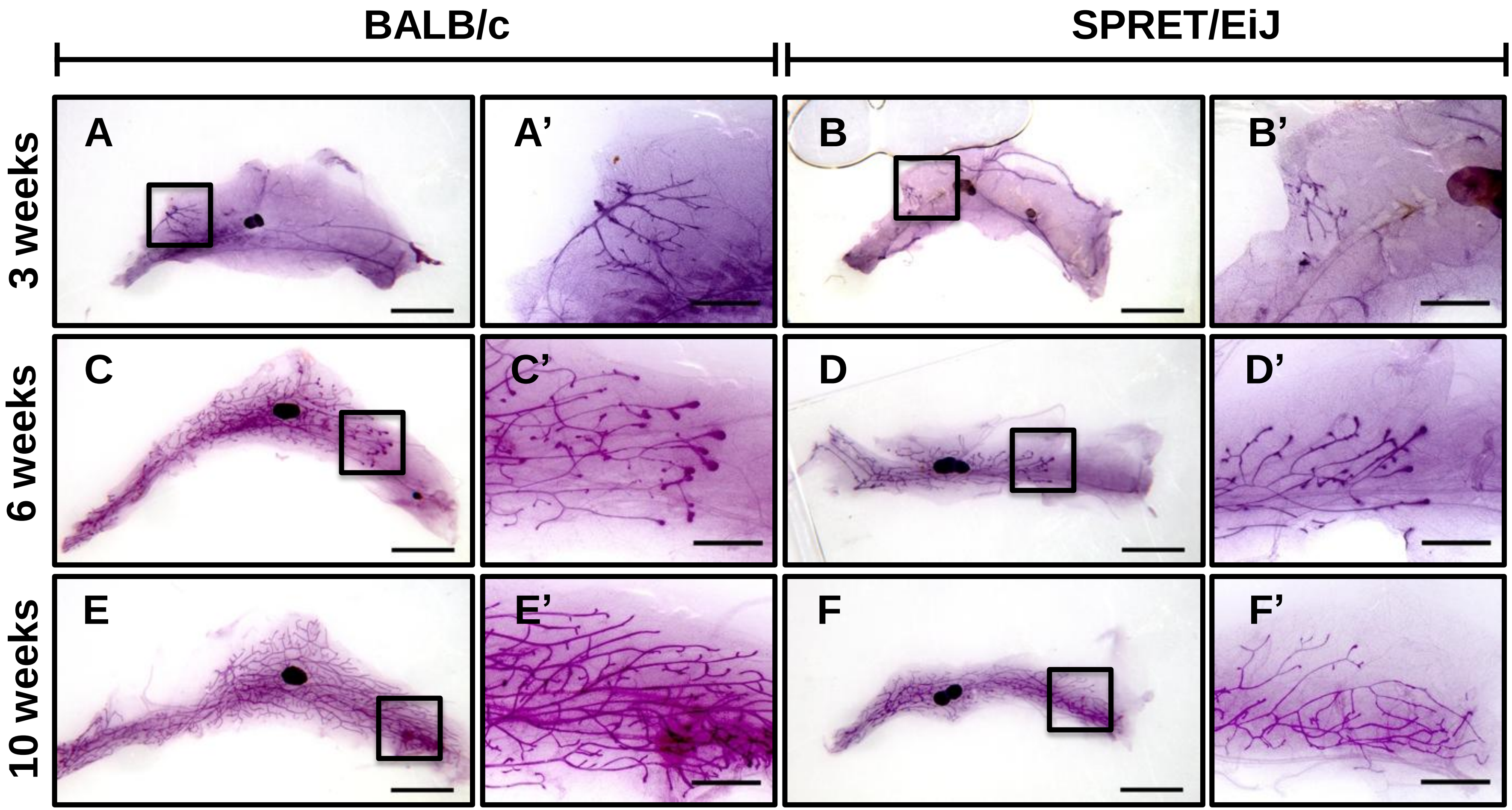

Figure S4. SPRET/EiJ mammary glands have a decrease in pubertal ductal outgrowth and ductal branching in comparison to BALB/c.

Whole-mount analysis of inguinal (no. 4) mammary glands from BALB/c and SPRET/EiJ at different stages of mammary gland development. Pre-pubertal (3 weeks; **A, A’, B, B’**), early pubertal (6 weeks; **C, C’, D, D’**), and late pubertal (10 weeks; **E, E’, F, F’**). Scale bars represent 1cm (**A, B, C, D, E, F**); 1mm (**A’, B’, C’, D’, E’, F’**)

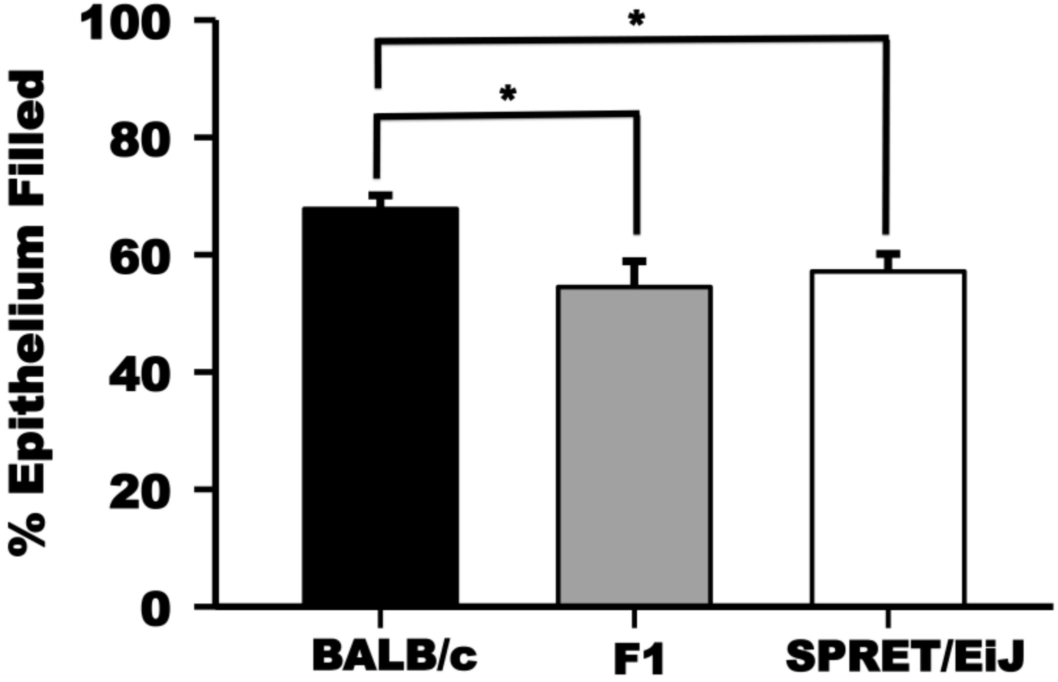

Figure S5. BALB/c mice exhibit an increase in mammary ductal outgrowth at late puberty in comparison to SPRET/EiJ and F1 mice.

Quantification of epithelium outgrowth from whole-mounts of inguinal (no. 4) mammary glands from BALB/c, F1, and SPRET/EiJ at 10 weeks. * indicates $p<0.05$. The p-values were obtained from t-test.

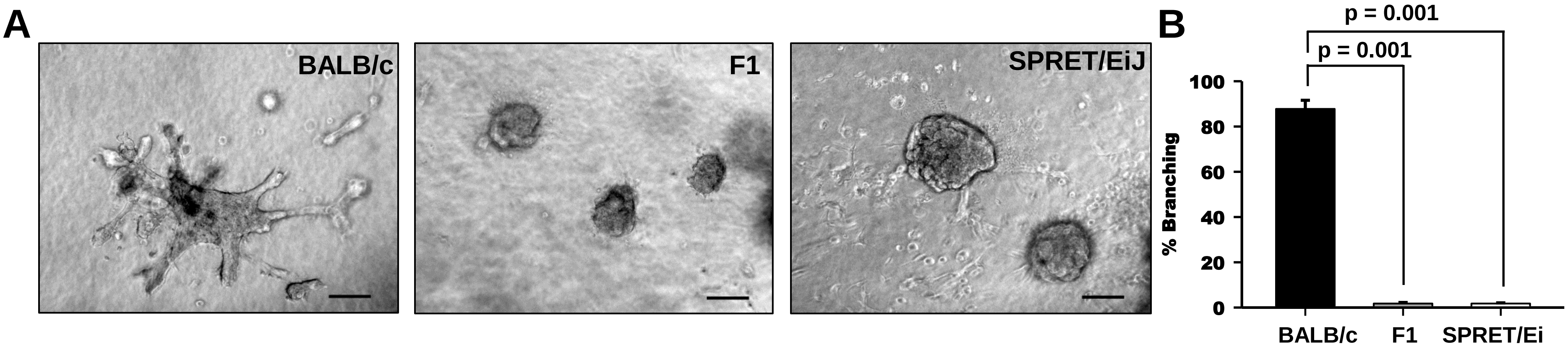

Figure S6. Invasion/branching in collagen-1 gels reveals SPRET/EiJ phenotype is not matrix dependent.

(A) A 3D organotypic culture model of mammary organoid cell invasion/branching. Mammary organoids from BALB/c, F1, or SPRET/EiJ mice were induced to branch by addition of 9nM TGFαk for 5 days in 3mg/ml collagen-1. **(B)** Invasion/branching of organoids were socred as positive when displaying three or more branches with lengths of at least half the diameter of the central organoid body. Percentage of cell invasion/branching of BALB/c, F1, and SPRET/EiJ. The p-values were obtained from t-test.

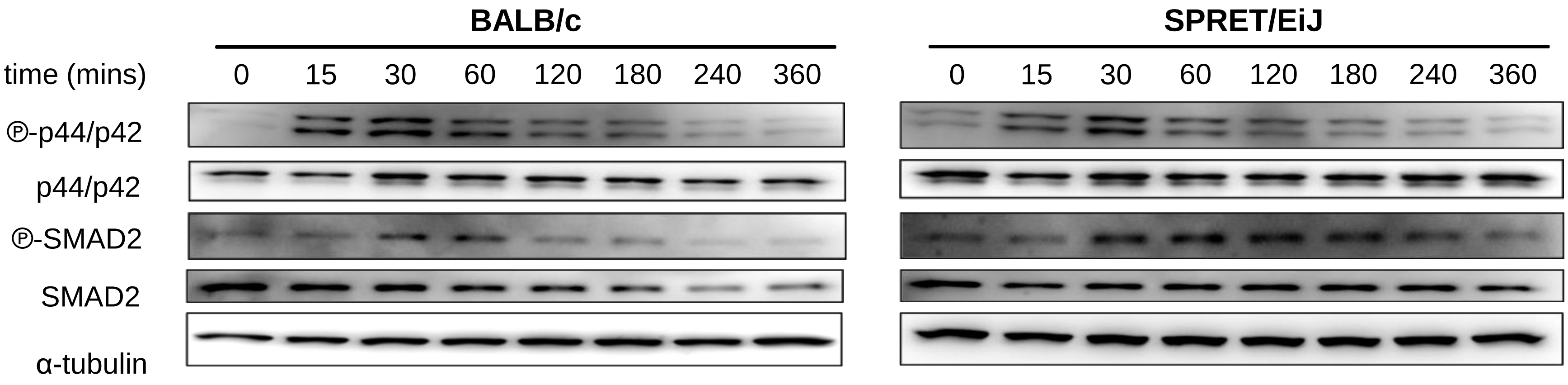

Figure S7. TGFα-induced MAPK^{ERK1,2} activation in SPRET/EiJ activates SMAD2 pathway.

Activation of MAPK^{ERK1,2} (P-p44, P-p42) levels are sustained for 1 hour in organoids stimulated with TGFα in both BALB/c and SPRET/EiJ but activation of SMAD2 (P-SMAD2) is sustained longer in SPRET/EiJ.

Table S1. List of candidate genes within genetic loci and their homologs in human

Mouse Chromosome	Mouse SNP	SNP location (bp)	Close to reported mouse tumor susceptible loci	Mouse gene name	Human gene name
1	rs13476248	174151673	Sluc5 and Scc3	Rgs4	RGS4
				1700084C01Rik	C1orf110
				Hsd17b7	HSD17B7
				Ddr2	DDR2
				Uap1	UAP1
				Uhmk1	UHMK1
				Sh2d1b1	SH2D1B
				EG665574	C1orf226
				1700015E13Rik	C1orf111
				Nos1ap	NOS1AP
				Olfml2b	OLFML2B
				Atf6	ATF6
				Dusp12	DUSP12
				Fcrlb	FCRLB
				Fcrla	FCRLA
				LOC100040778	LOC727947
				Fcgr2b	FCGR2B
				Fcgr4	FCGR3A
				Fcgr3	FCGR2A
				1700009P17Rik	C1orf192
				Sdhc	SDHC
				Mpz	MPZ
				Nr1i3	NR1I3
				Tomm40l	TOMM40L
				Apoa2	APOA2
				Fcer1g	FCER1G
				Ndufs2	NDUFS2
				Adamts4	ADAMTS4
				B4galt3	B4GALT3
				Ppox	PPOX
				Usp21	USP21
				Ufc1	UFC1
				Dedd	DEDD
				Nit1	NIT1
				Pfdn2	PFDN2
				1190002J23Rik	KLHDC9
				Pvrl4	PVRL4
				Arhgap30	ARHGAP30
				Usf1	USF1
				EG226654	KAT
				F11r	F11R
				Itlna	ITLN1
				Cd244	CD244
				Ly9	LY9
				Slamf7	SLAMF7
				Cd48	CD48

				LOC100041098	CCDC72
				Slamf1	SLAMF1
				Cd84	CD84
				Slamf6	SLAMF6
				Vangl2	VANGL2
				Nhlh1	NHLH1
				Ncstn	NCSTN
				Copa	COPA
				Pex19	PEX19
				Wdr42a	WDR42A
				Pea15a	PEA15
				Casq1	CASQ1
				Atp1a2	ATP1A2
				Igsf8	IGSF8
				Kcnj9	KCNJ9
				Kcnj10	KCNJ10
				Pigm	PIGM
				Slamf9	SLAMF9
				Igsf9	IGSF9
				Tagln2	TAGLN2
				Ccdc19	CCDC19
				EG240916	VSIG8
				Slamf8	SLAMF8
				Fcrl6	FCRL6
				Dusp23	DUSP23
				Crp	CRP
				Apcs	APCS
				Olfr16	OR10J5
				Olfr1408	OR10J1
				Olfr1404	OR10J3
				Fcer1a	FCER1A
				Darc	DARC
				Cadm3	CADM3
				Aim2	AIM2
				Olfr430	OR6N2
				Olfr429	OR6N1
				Olfr244	OR6K6
				Olfr421	OR6K3
				Olfr420	OR6K2
				Spna1	SPTA1
				Olfr419	OR10Z1
				1810030J14Rik	LOC649458
				Olfr417	OR10X1
				Olfr220	OR6Y1
				Grem2	GREM2
2	rs6376291	154141285	Bts1, Gct8, Hcs4, and Skts13	Rspo4	RSPO4
				Angpt4	ANGPT4
				5430432M24Rik	FAM110A
				2310046K01Rik	C20orf54

				Scrt2	SCRT2
				Srxn1	SRXN1
				Tcf15	TCF15
				Tbc1d20	TBC1D20
				Rbck1	RBCK1
				Trib3	TRIB3
				Nrsn2	NRSN2
				Sox12	SOX12
				Zcchc3	ZCCHC3
				6820408C15Rik	C20orf96
				EG629114	DEFB129
				Defb29	DEFB116
				Defb19	DEFB119
				Defb36	DEFB123
				OTTMUSG00000015862	DEFB124
				Rem1	REM1
				H13	HM13
				Id1	ID1
				Cox4i2	COX4I2
				Bcl2l1	BCL2L1
				Tpx2	TPX2
				Mylk2	MYLK2
				Fkhl18	FOXS1
				Dusp15	DUSP15
				Ttll9	TTLL9
				Pdrg1	PDRG1
				Xkr7	XKR7
				Hck	HCK
				Tm9sf4	TM9SF4
				Plagl2	PLAGL2
				Pofut1	POFUT1
				Kif3b	KIF3B
				Asxl1	ASXL1
				Commd7	COMMD7
				Dnmt3b	DNMT3B
				Mapre1	MAPRE1
				LOC100043869	TOMM20
				Spag4l	SPAG4L
				Bpil1	BPIL1
				Bpil3	BPIL3
				Rya3	C20orf185
				Psp	C20orf70
				1700058C13Rik	C20orf71
				Plunc	PLUNC
				U46068	C20orf114
				Cdk5rap1	CDK5RAP1
				Snta1	SNTA1
				Cbfa2t2	CBFA2T2
				Apba2bp	NECAB3
				E2f1	E2F1

				Pxmp4	PXMP4
				Zfp341	ZNF341
				Chmp4b	CHMP4B
				Raly	RALY
				Eif2s2	EIF2S2
				Asip	ASIP
				Ahcy	AHCY
				Itch	ITCH
				Dynlrb1	DYNLRB1
				Map1lc3a	MAP1LC3A
				Cdc91l1	PIGU
				Trp53inp2	TP53INP2
				Ncoa6	NCOA6
				Ggtl3	GGT7
				Acss2	ACSS2
				Gss	GSS
				Trpc4ap	TRPC4AP
				Edem2	EDEM2
				Procr	PROCR
				Mmp24	MMP24
				Itgb4bp	EIF6
				5530400B04Rik	FAM83C
				2410003P15Rik	UQCC
				Gdf5	GDF5
				Cep250	CEP250
				Ergic3	ERGIC3
				Cpne1	CPNE1
				Rbm12	RBM12
				Nfs1	NFS1
				2010100O12Rik	ROMO1
				Rbm39	RBM39
				Phf20	PHF20
				Scand1	SCAND1
				LOC668941	RPL37A
				0610011L14Rik	C20orf4
				Dlgap4	DLGAP4
				Tgif2	TGIF2
				1110008F13Rik	C20orf24
				Sla2	SLA2
3	rs6371982	34135438	Lscc2	Zmat3	ZMAT3
				Pik3ca	PIK3CA
				Kcnmb3	KCNMB3
				Zfp639	ZNF639
				Mfn1	MFN1
				Gnb4	GNB4
				Actl6a	ACTL6A
				Mrpl47	MRPL47
				Ndufb5	NDUFB5
				Usp13	USP13
				Pex2	PEX5L

				Ttc14	TTC14
				Ccdc39	CCDC39
				LOC100040232	CARHSP1
				Fxr1h	FXR1
				Dnajc19	DNAJC19
				Sox2	SOX2
				Atp11b	ATP11B
				Dcun1d1	DCUN1D1
				Mccc1	MCCC1
				Acad9	ACAD9
				Gpr103	QRFPR
				Anxa5	ANXA5
				Exosc9	EXOSC9
				Ccna2	CCNA2
				Bbs7	BBS7
				Trpc3	TRPC3
				4932438A13Rik	KIAA1109
				Tenr	ADAD1
				Il2	IL2
				Il21	IL21
3	rs3657112	148365541	Scc7	Mcoln3	MCOLN3
				Mcoln2	MCOLN2
				Edg7	LPAR3
				Ssx2ip	SSX2IP
				Ctbs	CTBS
				Spata1	SPATA1
				Bxdc5	BXDC5
				Dnase2b	DNASE2B
				Prkacb	PRKACB
				Ttll7	TTLL7
				Lphn2	LPHN2
				Eltd1	ELTD1
				Ifi44	IFI44
				H28	IFI44L
				Ptgfr	PTGFR
4	rs13477549	8803713		Nsmaf	NSMAF
				Tox	TOX
				Car8	CA8
				Rab2	RAB2A
				Chd7	CHD7
				4933402J24Rik	RLBP1L1
				Asph	ASPH
				Gdf6	GDF6
				2610301B20Rik	C8orf37
				Plekhf2	PLEKHF2
				2310030N02Rik	C8orf38
				Trp53inp1	TP53INP1
				Ccne2	CCNE2
				Ints8	INTS8
				Dpy19l4	DPY19L4

5	CEL-5_93652588	93652588	Hcs5 and Skts4	Epgn	EPGN
				Ereg	EREG
				Areg	AREGB
				Btc	BTC
				9130213B05Rik	DKFZP564O0823
				Rchy1	RCHY1
				Gm1045	C4orf26
				Cdkl2	CDKL2
				G3bp2	G3BP2
				Vdp	USO1
				Ppef2	PPEF2
				Asahl	NAAA
				Sdad1	SDAD1
				Cxcl9	CXCL9
				Cxcl10	CXCL10
				Cxcl11	CXCL11
				Art3	ART3
				Nup54	NUP54
				Scarb2	SCARB2
				D5Ertd593e	STBD1
				4932413O14Rik	CCDC158
				Shroom3	SHROOM3
				Ankrd56	ANKRD56
				Sept11	SEPT11
				Ccni	CCNI
				Ccng2	CCNG2
				Cxcl13	CXCL13
				Cnot6l	CNOT6L
				Mrpl1	MRPL1
				Fras1	FRAS1
7	rs13479513	127108163	Scc12, Sluc19 and Skts2	Jmjd5	JMJD5
				Nsmce1	NSMCE1
				Il4ra	IL4R
				Il21r	IL21R
				Gtf3c1	GTF3C1
				D430042O09Rik	KIAA0556
				Gsg1l	GSG1L
				Xpo6	XPO6
				Sbk1	SBK1
				Lat	LAT
				2210013K02Rik	SPNS1
				Nfatc2ip	NFATC2IP
				Cd19	CD19
				Rabep2	RABEP2
				Atp2a1	ATP2A1
				Sh2b1	SH2B1
				Tufm	TUFM
				Atxn2l	ATXN2L
				Eif3s8	EIF3CL

				Cln3	CLN3
				Apob48r	APOB48R
				Il27	IL27
				Nupr1	NUPR1
				Ccdc101	CCDC101
				Sult1a1	SULT1A1
				Giyd2	GIYD2
				Coro1a	CORO1A
				Mapk3	MAPK3
				Gdpd3	GDPD3
				Ypel3	YPEL3
				Tbx6	TBX6
				Ppp4c	PPP4C
				Aldoa	ALDOA
				1500016O10Rik	FAM57B
				4930451I11Rik	C16orf92
				Doc2a	DOC2A
				Ccdc95	INO80E
				Hirip3	HIRIP3
				1110032O16Rik	TMEM219
				Kctd13	KCTD13
				Sez6l2	SEZ6L2
				Cdipt	CDIPT
				Mvp	MVP
				2900092E17Rik	C16orf53
				Maz	MAZ
				Kif22	KIF22
				1810010M01Rik	ZG16
				AI467606	C16orf54
				Qprt	QPRT
				Spn	SPN
				Cd2bp2	CD2BP2
				Tbc1d10b	TBC1D10B
				Mylpf	MYLPF
				41883	41883
				Zfp553	ZNF48
				Zfp771	ZNF771
				2410015N17Rik	DCTPP1
				Sephs2	SEPHS2
				Itgal	ITGAL
				Zfp768	ZNF768
				Zfp764	ZNF764
				Zfp688	ZNF688
				Zfp689	ZNF689
				Prr14	PRR14
				Fbs1	FBRS
				LOC100043597	SRCAP
				Phkg2	PHKG2
				Gm166	C16orf93
				Rnf40	RNF40

				Zfp629	ZNF629
				Bcl7c	BCL7C
				Ctf1	CTF1
				Fbxl19	FBXL19
				Tmem142c	ORAI3
				Setd1a	SETD1A
				Hsd3b7	HSD3B7
				Stx1b2	STX1B
				Stx4a	STX4
				Zfp668	ZNF668
				Zfp646	ZNF646
				BC039632	POL3S
				Vkorc1	VKORC1
				Bckdk	BCKDK
				Myst1	MYST1
				Prss8	PRSS8
				Prss36	PRSS36
				Fus	FUS
				Pycard	PYCARD
				Trim72	TRIM72
				Itgam	ITGAM
				Itgax	ITGAX
				Itgad	ITGAD
				Cox6a2	COX6A2
				Armc5	ARMC5
				Tgfb1i1	TGFB1I1
				Slc5a2	SLC5A2
				BC017158	C16orf58
				Rgs10	RGS10
				Tial1	TIAL1
				Bag3	BAG3
				Inpp5f	INPP5F
				1110007A13Rik	C10orf119
				Sec23ip	SEC23IP
				Ppapdc1a	PPAPDC1A
				Brwd2	BRWD2
				Fgfr2	FGFR2
				Ate1	ATE1
				Nsmce4a	NSMCE4A
				Tacc2	TACC2
8	rs3685424	57763292	Sluc9	Adam29	ADAM29
				Glra3	GLRA3
				Hpgd	HPGD
				BC088983	KIAA1712
				Fbxo8	FBXO8
				Hand2	HAND2
				Scrg1	SCRG1
				Sap30	SAP30
				Hmgb2	HMGB2
				Galnt7	GALNT7

				Aadat	AADAT
				Mfap3l	MFAP3L
				2700029M09Rik	C4orf27
				Clcn3	CLCN3
				Nek1	NEK1
9	rs13480208	55192291	Elads3	Ddx10	DDX10
				Exph5	EXPH5
				Kdelc2	KDELC2
				4930550C14Rik	C11orf65
				Atm	ATM
				Npat	NPAT
				Acat1	ACAT1
				Cul5	CUL5
				Rab39	RAB39
				Slc35f2	SLC35F2
				Elmod1	ELMOD1
				Tnfaip8l3	TNFAIP8L3
				Cyp19a1	CYP19A1
				Gldn	GLDN
				Dmxl2	DMXL2
				4933412E14Rik	SH2D7
				Cib2	CIB2
				Idh3a	IDH3A
				Acsbg1	ACSBG1
				Dnaja4	DNAJA4
				Wdr61	WDR61
				Crabp1	CRABP1
				Ireb2	IREB2
				C630028N24Rik	LOC123688
				Psma4	PSMA4
				Chrna5	CHRNA5
				Chrna3	CHRNA3
				Chrnb4	CHRNB4
				Ube2q2	UBE2Q2
				Fbxo22	FBXO22
				Nrg4	NRG4
				AI118078	C15orf27
				Etfa	ETFA
				Isl2	ISL2
				Zfp291	SCAPER
				Rcn2	RCN2
				Pstpip1	PSTPIP1
				Tspan3	TSPAN3
				C230081A13Rik	SGK269
				Hmg20a	HMG20A
				LOC100039535	UBE2S
				Lingo1	LINGO1
				Odf3l1	ODF3L1
				Cspg4	CSPG4
				Sh3px3	SNX33

				Imp3	IMP3
				Snupn	SNUPN
				Ptpn9	PTPN9
				Sin3a	SIN3A
				Man2c1	MAN2C1
				Neil1	NEIL1
				Commd4	COMMD4
				Trcg1	LOC730036
				1700017B05Rik	C15orf39
				Ppcdc	PPCDC
				Scamp5	SCAMP5
				Rpp25	RPP25
				Cox5a	COX5A
				2310046O06Rik	C15orf17
				Mpi1	MPI
				Scamp2	SCAMP2
				Ulk3	ULK3
				Cplx3	CPLX3
				Lman1l	LMAN1L
				Csk	CSK
				Cyp1a2	CYP1A2
				Cyp1a1	CYP1A1
				Edc3	EDC3
				Clk3	CLK3
				Arid3b	ARID3B
				Ubl7	UBL7
				Sema7a	SEMA7A
				Cyp11a1	CYP11A1
				4930535E21Rik	CCDC33
				Stra6	STRA6
11	rs13481099	73722822	Scc15 and Sluc4/Pas5	Aipl1	AIPL1
				6720460F02Rik	FAM64A
				Pitpnm3	PITPNM3
				4933427D14Rik	KIAA0753
				Txnl5	TXNDC17
				Med31	MED31
				4930563E22Rik	C17orf100
				Slc13a5	SLC13A5
				LOC628100	FBXO39
				Tekt1	TEKT1
				D130058I21Rik	SMTNL2
				Ggt6	GGT6
				Mybbp1a	MYBBP1A
				9830002I17Rik	SPNS3
				Ube2g1	UBE2G1
				Ankfy1	ANKFY1
				Cyb5d2	CYB5D2
				Zzef1	ZZEF1
				Atp2a3	ATP2A3

				P2rx1	P2RX1
				Camkk1	CAMKK1
				1200014J11Rik	C17orf85
				Itgae	ITGAE
				Gsg2	GSG2
				P2rx5	P2RX5
				Tmem93	TMEM93
				Ctns	CTNS
				Carkl	SHPK
				Trpv1	TRPV1
				Trpv3	TRPV3
				Aspa	ASPA
				Spata22	SPATA22
				Olfr20	OR1E1
				Olfr376	OR1E2
				1700012C15Rik	ZNF679
				OTTMUSG00000006163	ZNF616
				Olfr402	OR3A2
				Olfr43	OR1A1
				Olfr410	OR3A1
				Olfr411	OR3A3
				Olfr412	OR1D2
				Garnl4	GARNL4
				1300001I01Rik	KIAA0664
				Pafah1b1	PAFAH1B1
				Mett10d	METT10D
				Mnt	MNT
				Rutbc1	SGSM2
				Tsr1	TSR1
				Srr	SRR
				Smg6	SMG6
				LOC100043429	SUMO2
				Hic1	HIC1
				Ovca2	OVCA2
				Dph1	DPH1
				Rtn4rl1	RTN4RL1
				Rpa1	RPA1
				Smyd4	SMYD4
				Serpinf1	SERPINF1
				Serpinf2	SERPINF2
				Wdr81	WDR81
				2010305C02Rik	TLCD2
				Prpf8	PRPF8
				Rilp	RILP
				Scarf1	SCARF1
				Slc43a2	SLC43A2
				Pitpna	PITPNA
				Pps	INPP5K
				Myo1c	MYO1C
				Crk	CRK

				Ywhae	YWHAE
				Doc2b	DOC2B
				Rph3al	RPH3AL
				1700016K19Rik	C17orf97
				1500005K14Rik	FAM101B
				Vps53	VPS53
12	rs3700012	101759668	Sluc12 and Hcc3	Ches1	FOXN3
				2610021K21Rik	C14orf143
				Tdp1	TDP1
				Kcnk13	KCNK13
				Psmc1	PSMC1
				BC002230	C14orf102
				Ttc7b	TTC7B
				Rps6ka5	RPS6KA5
				9030617O03Rik	C14orf159
				Gpr68	GPR68
				0610010D24Rik	CCDC88C
				Smek1	SMEK1
				4930463M05Rik	KIF4B
				4932415G16Rik	CATSPERB
				Mtac2d1	TC2N
				Fbln5	FBLN5
				Trip11	TRIP11
				Atxn3	ATXN3
				Cpsf2	CPSF2
				Slc24a4	SLC24A4
				Rin3	RIN3
				Lgmn	LGMN
				Golga5	GOLGA5
				Chga	CHGA
				Itpk1	ITPK1
				Moap1	MOAP1
				D230037D09Rik	C14orf109
				5730410I19Rik	UBR7
				Btbd7	BTBD7
				9030205A07Rik	KIAA1409
				Prima1	PRIMA1
				EG544888	FAM181A
				Asb2	ASB2
				Otub2	OTUB2
				Ddx24	DDX24
				D12Ertd647e	IFI27
				Ifi27	IFI27L2
				8430415E04Rik	PPP4R4
				Serpina10	SERPINA10
				Serpina6	SERPINA6
				Serpina1d	SERPINA1
				Serpina11	SERPINA11
				Serpina9	SERPINA9
				Serpina12	SERPINA12

				Serpina5	SERPINA5
				Serpina3n	SERPINA3
14	rs3708535	86838897		Pcdh17	PCDH17
				Diap3	DIAPH3
				Tdrd3	TDRD3
				Pcdh20	PCDH20
15	rs13482641	71917280	Sluc26	Zfat1	ZFAT
				Khdrbs3	KHDRBS3
				Col22a1	COL22A1
				Kcnk9	KCNK9
				1810044A24Rik	TRAPPC9
				Chrac1	CHRAC1
				Eif2c2	EIF2C2
				Ptk2	PTK2
				Dennd3	DENND3
				Slc45a4	SLC45A4
				Gpr20	GPR20
				pPtp4a3	PTP4A3
				Gm628	FLJ43860
16	rs4186129	50727848	Sluc27	Morc1	MORC1
				Trat1	TRAT1
				EG667501	TWISTNB
				Retnlg	RETNLB
				2310047C04Rik	DZIP3
				C330027C09Rik	KIAA1524
				Myh15	MYH15
				Ift57	IFT57
				Cd47	CD47
				Bbx	BBX
				Ccdc54	CCDC54
				Cblb	CBLB
				Alcam	ALCAM
17	rs13483012	48599852	Skts10	Vegfa	VEGFA
				Mrps18a	MRPS18A
				1700027N10Rik	RSPH9
				Mad2l1bp	MAD2L1BP
				Gtpbp2	GTPBP2
				Polh	POLH
				Xpo5	XPO5
				Rpo1-1	POLR1C
				Yipf3	YIPF3
				Gm88	C6orf154
				Tjap1	TJAP1
				Egfl9	DLK2
				Abcc10	ABCC10
				Zfp318	ZNF318
				Crip3	CRIP3
				Slc22a7	SLC22A7
				Ttbk1	TTBK1
				BC048355	C6orf108

				Parc	CUL9
				Srf	SRF
				Ptk7	PTK7
				Klc4	KLC4
				Mrpl2	MRPL2
				Cul7	CUL7
				BC011248	C6orf153
				Klhdc3	KLHDC3
				Mea1	MEA1
				Ppp2r5d	PPP2R5D
				Pex6	PEX6
				Gnmt	GNMT
				Tnrc5	CNPY3
				Ptcra	PTCRA
				Rpl7l1	RPL7L1
				BC032203	KIAA0240
				Tbcc	TBCC
				Prph2	PRPH2
				Ubr2	UBR2
				Trerf1	TRERF1
				Mrps10	MRPS10
				Guca1b	GUCA1B
				Guca1a	GUCA1A
				EG545208	MRFAP1
				AI661453	C6orf132
				Tbn	TAF8
				Ccnd3	CCND3
				Bysl	BYSL
				Trfp	MED20
				Usp49	USP49
				1110002E23Rik	TOMM6
				Frs3	FRS3
				Pgc	PGC
				Tcfeb	TFEB
				Foxp4	FOXP4
				Trem1	TREM1
				Treml4	TREML4
				Treml2	TREML2
				Trem2	TREM2
				Treml1	TREML1
				Nfya	NFYA
				AI314976	C6orf130
				Apobec2	APOBEC2
				Bzrpl1	BZRPL1
				Unc5cl	UNC5CL
				Lrfn2	LRFN2
				Mocs1	MOCS1
				Daam2	DAAM2
				Kif6	KIF6
				Rftn1	RFTN1

				Dazl	DAZ2
				Plcl2	PLCL2
				Tbc1d5	TBC1D5
